# Clinical significance of TLR3 and TLR4 in peripheral blood mononuclear cells from children with Henoch-Schönlein purpura nephritis

**DOI:** 10.3892/etm.2014.1638

**Published:** 2014-03-27

**Authors:** HONG CHANG, DONG-SHENG YU, XIU-QIN LIU, QIU-YE ZHANG, NA CHENG, SHOU-QING ZHANG, ZHENG-HAI QU

**Affiliations:** 1 Department of Pediatrics, The Affiliated Hospital of Qingdao University Medical College, Qingdao, Shandong 266003, P.R. China; 2 Department of Interventional Medicine, The Affiliated Hospital of Qingdao University Medical College, Qingdao, Shandong 266003, P.R. China; 3 Department of Pediatrics, Municipal Hospital of Qingdao, Qingdao, Shandong 266071, P.R. China

**Keywords:** purpura, allergy, Henoch-Schönlein purpura nephritis, Toll-like receptor

## Abstract

The aim of the present study was to investigate the expression levels and clinical significance of Toll-like receptor (TLR) 3 and 4 in peripheral blood mononuclear cells (PBMCs) collected from children with Henoch-Schönlein purpura (HSP) nephritis. The randomized controlled trial was conducted between August 2011 and March 2013, and 105 children with a clinical diagnosis of HSP were enrolled in the study. According to the 24-h urinary protein measurements and the presence of renal damage, the 105 cases were divided into groups A, B and C as follows: Group A, children with HSP but without renal damage; group B, children with HSP nephritis but without proteinuria; group C, children with HSP nephritis and proteinuria. A total of 30 healthy children were enrolled in the normal control group (group N). The primary endpoints were the detection of TLR3 and 4 mRNA and protein expression levels in PBMCs by flow cytometry and quantitative polymerase chain reaction. The mRNA and protein expression levels of TLR4 in the PBMCs were significantly higher in groups A, B and C when compared with group N. In addition, the mRNA and protein expression levels of TLR4 in group C were much higher when compared with groups A and B. A positive correlation was identified between TLR4 protein expression and 24-h urinary protein levels in group C. The expression levels of TLR3 did not significantly differ among the groups. Protein and mRNA expression levels of TLR4 in PBMCs significantly increased and exhibited a positive correlation with urinary protein excretion. These results indicate that aberrant activation of TLR4 may be relevant to the development of HSP nephritis.

## Introduction

Secondary kidney damage is the most common complication in children with Henoch-Schönlein purpura (HSP) nephritis. Currently, HSP is often considered to be preceded by an infection (1,2); however, the cause of immunity function disorder remains unclear (3). Toll-like receptors (TLRs) are a class of important pattern recognition receptors, which function as a bridge, connecting inherent and adaptive immunities (4).

Allergic purpura is one of the most common types of vasculitis in children and mainly affects the vessels of the skin, gastrointestinal tract and kidneys. Approximately 20–60% of children with allergic purpura are complicated with renal damage, which is an important factor affecting the prognosis. Infection is the most important risk factor of HSP nephritis, of which the incidence is >70% (1,2). Group A Streptococcus is the most common precipitant, demonstrable in up to one-third of cases, but exposure to Bartonella, *Haemophilus parainfluenza* and numerous vaccines and drugs may precede the development of HSP. Pathogens may activate an abnormal immune response via a number of methods. The role of microbial antigens in the pathogenesis of HSP remains elusive.

Human TLRs are a class of transmembrane receptors that induce signaling pathways. TLRs are the first line of defense for the host to initiate an immune and inflammatory response. Through recognition of pathogen-associated molecular patterns (PAMPs) in pathogenic organisms, TLRs activate intracellular signaling pathways, resulting in the release of a series of inflammatory cytokines and the initiation of an adaptive immune response. In addition, TLRs function as a bridge, connecting inherent immunity and adaptive immunity (4). TLRs are expressed in immunocytes, including monocytes, macrophages and dendritic cells, as well as in renal cells. Abnormal expression of TLRs may cause numerous kidney diseases, including interstitial nephritis, immune complex nephritis, renal ischemia-reperfusion injury and rejection of renal transplantation (5–8). However, whether expression of TLRs is associated with the development of HSP nephritis remains unclear.

The aim of the present study was to detect the expression levels of TLRs in PBMCs from children with HSP nephritis and analyze urinary protein excretion to explore the effects of TLRs on the pathogenesis of HSP nephritis.

## Subjects and methods

### Subjects and groups

Between August 2011 and March 2013, 105 children aged between 2 and 14 years-old were diagnosed with acute HSP, on the basis of EULAR/PReS criteria for the classification of childhood vasculitis in 2005 (9). The subjects included 50 males and 55 females with an average age of 6 years. The children initially presented with HSP and had not been administered glucocorticoids, immunosuppressors or heparin in the 4 weeks prior to disease occurrence. According to the 24-h urinary protein measurements and the presence of renal damage, 105 cases were divided into groups A, B and C as follows: Group A, 57 children with HSP but without kidney damage; group B, 25 children with HSP nephritis but no proteinuria; and group C, 23 children with HSP nephritis and proteinuria. An additional 30 healthy children in The Affiliated Hospital of Qingdao University Medical College (Qingdao, China) were recruited for the normal control group (group N), which included 16 males and 14 females with an average age of 6.4 years-old (range, 3–12 years). These individuals had no anaphylactic disease history prior to the study. The blood samples were detected simultaneously. This study was conducted in accordance with the Declaration of Helsinki and with approval from the Ethics Committee of the Affiliated Hospital of Qingdao University Medical College (Qingdao, China). Written informed consent was obtained from patients or their families.

### Isolation of PBMCs

Blood samples (2–3 ml) were obtained under sterile conditions and PBMCs were isolated with lymphocyte separation medium through density gradient centrifugation. Next, 1 ml RNAiso Plus (Takara Biotechnology, Co., Ltd., Dalian, China) was added and the samples were stored at −80°C.

### cDNA synthesis

Total RNA was extracted from PBMCs with Takara reagent, according to the manufacturer’s instructions (Takara Biotechnology, Co., Ltd.). The concentration of total RNA was detected using an ultraviolet spectrophotometer. Quantitative polymerase chain reaction (qPCR) was performed with TLR3 and TLR4 primers [Table I; Sangon Biotech (Shanghai) Co., Ltd., Shanghai, China]. Optimal reaction conditions were 35–40 cycles of a two-stage PCR. The amplified PCR products of TLR3 and TLR4 were resolved by 2% agarose gel electrophoresis, and recycled. The products were sequenced by Sangon Biotech (Shanghai) Co., Ltd. and the sequencing results were identical to those from GenBank.

### qPCR

First strand cDNA was amplified using a qPCR thermal cycler (ABI7000; Applied Biosystems, Inc., Foster City, CA, USA). For the relative comparison of each gene expression, the qPCR data were analyzed using the 2^−ΔΔCT^ method (10). The Ct value of the endogenous control (GAPDH) was subtracted from the Ct value of each target gene to normalize the quantity of sample cDNA added to each reaction.

### Flow cytometry (FCM)

Peripheral blood (400 μl) was added to four marked tubes (100 μl/tube) and 5 μl anti-CD14^+^-fluorescein isothiocyanate-conjugated antibody was added. The first tube included 5 μl isotype control of anti-TLR4-phycoerythrin (PE) antibody (mouse IgG2A marked by PE; K isotype), the second tube contained 5 μl anti-TLR4-PE antibody, the third tube included 5 μl isotype control of anti-TLR3 antibody (mouse IgG1; K isotype) and the fourth tube contained 5 μl anti-TLR3-PE antibody. Samples were then stored at room temperature in the dark for 15 min. The tubes were centrifuged for 5 min (626 × g) and washed with phosphate-buffered saline. Finally, the expression levels of TLR3 and TLR4 from CD14^+^ monocytes were measured by FCM (Beckman Coulter, Miami, FL, USA). CellQuest software (BD Biosciences, San Jose, CA, USA) was used to analyze the results. All antibodies were purchased from eBioscience (San Diego, CA, USA).

### Statistical analysis

Statistical analysis was performed using SPSS 17.0 software (SPSS, Inc., Chicago, IL, USA) and the results are presented as the mean ± SEM. One-way analysis of variance was used for multiple-group comparisons, after establishing that the data in the groups were normally and equally distributed. The least significant difference t-test was used for two-sample comparisons from multiple groups, after establishing that the data in the groups were not equally distributed. Pearson analysis was used to analyze correlations between the groups. P<0.05 was considered to indicate a statistically significant difference.

## Results

### Expression of TLR3 and TLR4

**Figure 1:**
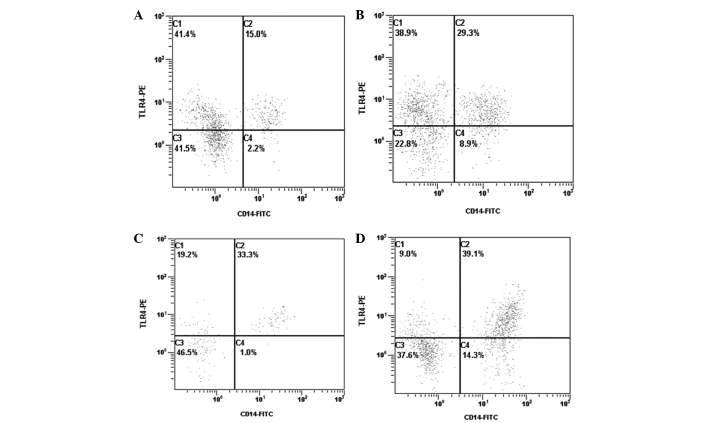
Expression of TLR4 protein in PBMCs. Protein expression of TLR4 from PBMCs in (A) group N, (B) group A, (C) group B and (D) group C. TLR, Toll-like receptor; PBMCs, peripheral blood mononuclear cells.

mRNA expression levels of TLR4 were significantly higher in groups A, B and C when compared with group N (P<0.05, <0.01 and <0.01, respectively). Protein expression levels of TLR4 were also significantly higher in groups A, B and C when compared with group N (P<0.01, <0.01 and <0.01, respectively). In addition, mRNA and protein expression levels of TLR4 in group C were markedly higher when compared with group A (P<0.01 and <0.05, respectively) and group B (P<0.05 and <0.01, respectively). There were no significant differences in TLR4 mRNA and protein expression levels between groups A and B (P>0.05 and >0.05, respectively; Table II; Fig. 1). In addition, the expression levels of TLR3 were not significantly different between each of the 4 groups (Table II).

**Figure 2:**
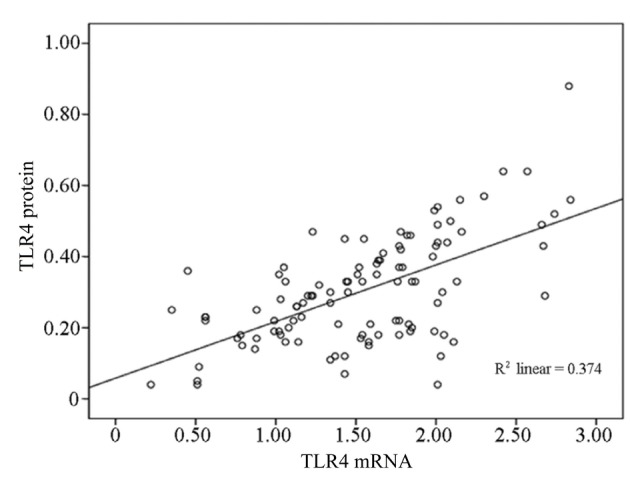
Correlation between mRNA and protein expression levels of TLR4. TLR, Toll-like receptor.

In addition, the mRNA expression levels of TLR4 (1.53±0.58, n=105) in all the patients with HSP (groups A, B, and C) exhibited a positive correlation with the level of TLR4 protein expression (0.30±0.17, n=105; r=0.61, P<0.01) (Fig. 2).

### Correlation analysis between TLR4 protein expression and 24-h proteinuria

**Figure 3:**
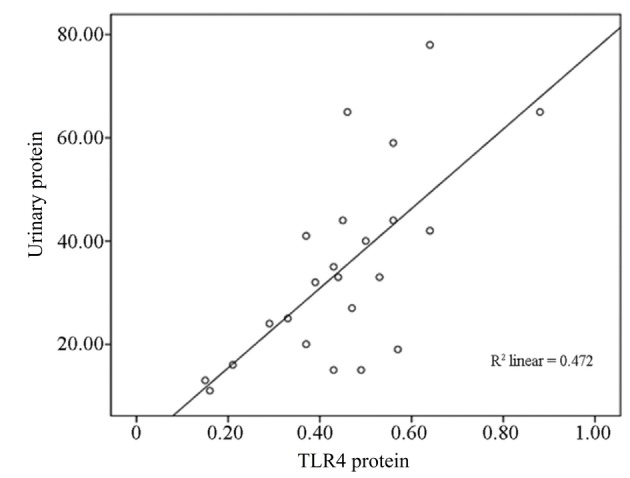
Correlation analysis between TLR4 protein expression levels and 24-h urinary albumin in group C. TLR, Toll-like receptor.

TLR4 protein expression in group C (HSP with proteinuria) and 24-h urinary protein (35±18 mg/kg) exhibited a positive correlation (r=0.69, P<0.01; Fig. 3) in PBMCs from children with HSP.

## Discussion

Anaphylactoid purpura (also known as HSP) is small-vessel vasculitis that involves the skin, connective tissues, scrotum, joints, gastrointestinal tract and kidneys. Secondary kidney damage is the most common complication in children with HSP nephritis, which directly affects the course and prognosis of HSP (1,2). However, the mechanism of action remains unclear, although it may include the disorder of humoral and cell immunities and cytokines or inflammatory mediators (3). A number of studies have indicated that HSP is often preceded by an infection. There is a close correlation between the incidence of HSP and infection. In particular, intractable HSP has a higher incidence of infection. However, the cause of immunity function disorder currently remains unclear.

TLRs are a class of important pattern recognition receptors involved in the innate immune system that can recognize PAMPs to activate cell signaling systems, generating a series of proinflammatory cytokines to activate the adaptive immune response (11–14). Activation of innate immunity is a critical step in the development of antigen-specific acquired immunity and individual receptors may be upregulated during infection and inflammation. Thus, we hypothesized that the incidence and development of HSP and HSP nephritis may be mediated by TLRs when patients are infected by bacteria or viruses.

TLRs are single membrane-spanning, non-catalytic receptors that are usually expressed in sentinel cells, including macrophages and dendritic cells. Exogenous ligands of TLRs are from PAMPs, including bacterial cell-surface lipopolysaccharides (LPSs), teichoic acid and nucleic acids of bacteria and viruses. Endogenous ligands include heat shock protein 60, fibrinogen and extracellular matrix components. TLR3 is able to recognize viral RNA and synthetic RNA, while TLR4 is able to recognize numerous ligands, including LPS, HSPs, heparin, fibrinogen and taxol. At present, 13 types of TLRs have been identified (TLR1-TLR13).

Although TLR signals are the first defense against infection from microbes, over-activated TLR signaling causes inflammatory cell infiltration to generate cytokines and autoantibodies, thus, inducing autoimmune diseases (15–18). TLR upregulation may be involved in renal disease and there is increasing data supporting a role for TLRs in infectious autoimmune and inflammatory disorders of the kidney (3). In a crescentic glomerulonephritis experiment, wild-type C57BL/6 mice and TLR-deficient mice were treated with the same dose of LPS. The wild-type mice developed severe glomerular injury, characterized by glomerular thrombosis, crescent formation and glomerular macrophage infiltration. This indicated that the TLR2 signaling pathway was involved in crescentic glomerulonephritis (19). Additional studies have also demonstrated that the expression of B7-1 was markedly increased following LPS treatment via the TLR4 signaling pathway, which causes proteinuria (20). Pawar *et al* reported that LPS may activate the TLR2 and TLR4 signaling pathway in a mouse model of lupus nephrosis, and promote the inflammatory factor secretion (21). This process leads to continuous kidney damage. Coppo *et al* (20) found that protein and gene expression levels of TLR4 increased in circulating mononuclear cells of patients with IgA nephropathy. This indicated that TLR4 signaling mediated the abnormal immunity of IgA nephropathy. Therefore, it was hypothesized that TLRs participate in the pathogenesis of HSP nephritis, however, the correlation between TLRs and HSP nephritis is yet to be studied.

The results of the present study demonstrated that mRNA and protein expression levels of TLR3 did not significantly differ between the normal control group and the other groups. However, the mRNA and protein expression levels of TLR4 in PBMCs were shown to be higher in groups A, B and C when compared with the control group. Furthermore, protein and mRNA expression levels of TLR4 were higher in group C than in groups A and B, and protein expression levels of TLR4 in group C exhibited a positive correlation with 24-h urinary protein excretions.

In conclusion, the mRNA and protein expression levels of TLR4 in PBMCs from HSP nephritis children were significantly increased, which positively correlated with the level of 24-h urinary protein. This was consistent with our previous hypothesis. The results indicated that activation of TLR4 signaling was associated with HSP and aberrant activation of TLR4 may cause kidney damage. Thus, downregulating TLR4 expression levels or blockading the TLR4 signaling pathway are candidate targets for future HSP nephritis treatments (22), although the exact mechanism of action requires further study. In the present study, the mRNA and protein expression levels of TLR3 and TLR4 in children with HSP nephritis were investigated only in PBMCs. Other TLRs and associated signaling factors have not yet been studied or TLR expression in renal tissue. Therefore, further study to elucidate the role of the innate immune system in renal injury is required.

## Figures and Tables

**Table I:** Primers of TLR3, TLR4 and GAPDH.

Gene	Sequences	Annealing temperature (°C)	Fragment size (bp)
TLR3	Sense: 5′-GTCCAACGGCTTTGACGAGAT-3′ Antisense: 5′-CTGGCCCGAAAACCTTCTTCT-3′	56	188
TLR4	Sense: 5′-TGTCCTCCCACTCCAGGTAAGT-3′ Antisense: 5′-GATTGCTCAGACCTGGCAGTT-3′	55	144
GAPDH	Sense: 5′-TCATGGGTGTGAACCATGAGAA-3′ Antisense: 5′-GGCATGGACTGTGGTCATGAG-3′	57	111

TLR, Toll-like receptor.

**Table II:** mRNA and protein expression levels of TLR3 and TLR4 (mean ± SEM).

Group	Cases	TLR3 mRNA	TLR3 protein	TLR4 mRNA	TLR4 protein
A	57	0.54±0.24	0.10±0.06	1.30±0.62^a^^b^	0.23±0.11^c^^d^
B	25	0.64±0.22	0.09±0.03	1.78±0.32^c^^d^	0.32±0.12^b^^c^
C	23	0.59±0.23	0.12±0.07	2.20±0.50^c^	0.45±0.16^c^
N	30	0.62±0.77	0.10±0.06	1.10±0.50	0.14±0.08
F-value		1.78	0.86	37.33	24.01

a P<0.05, vs. group N;

b P<0.01, vs. group C;

c P<0.01, vs. group N;

d P<0.05, vs. group C.

TLR, Toll-like receptor.
